# Ameliorative Effects of Herbal Combinations in Hyperlipidemia

**DOI:** 10.1155/2011/160408

**Published:** 2011-09-15

**Authors:** Nishant P. Visavadiya, A. V. R. L. Narasimhacharya

**Affiliations:** ^1^BRD School of Biosciences, Sardar Patel University, Sardar Patel Maidan, Vadtal Road, Satellite Campus, P.O. Box 39, Vallabh Vidyanagar 388 120, India; ^2^Spinal Cord and Brain Injury Research Center (SCoBIRC), University of Kentucky, Biomedical & Biological Sciences Research Building B0436-41, 741 S. Limestone St., Lexington, KY 40536, USA

## Abstract

The roots of *Glycyrrhiza glabra, Withania somnifera, Asparagus racemosus, and Chlorophytum borivilianum* and seeds of *Sesamum indicum* are ayurvedic medicinal plants used in India to treat several ailments. Our previous studies indicated that these plants possess hypolipidemic and antioxidant potential. The present study was aimed at investigating the composite effects of these plants on hypercholesterolemic rats. Three different combinations (5 gm%, given for four weeks) used in this study effectively reduced plasma and hepatic lipid profiles and increased fecal excretion of cholesterol, neutral sterol, and bile acid along with increasing the hepatic HMG-CoA reductase activity and bile acid content in hypercholesterolemic rats. Further, all three combinations also improved the hepatic antioxidant status (catalase, SOD, and ascorbic acid levels) and plasma total antioxidant capacity with reduced hepatic lipid peroxidation. Overall, combination I had the maximum effect on hypercholesterolemic rats followed by combinations II and III due to varying concentrations of the different classes of phytocomponents.

## 1. Introduction

Hypercholesterolemia is one of the major risk factors that precipitate coronary heart disease (CHD) and atherosclerosis [1]. Besides medication, composition of the diet also plays an important role in the management of lipid and lipoprotein concentrations in blood. Plant-based therapies are recognized for their therapeutic applications as they either have minimal or no side effects [1, 2]. Over the past 20 years or so, interest in traditional medicines has increased considerably in many parts of the world. Traditional medicines all over the world are being reevaluated by extensive research on different plant species with regard to their therapeutic principles and potential. Despite the progress in conventional chemistry and pharmacology in producing effective drugs, the plant kingdom might yet provide useful sources of new medicines. Plants produce an amazing variety of metabolites such as isoflavones, phytosterols, saponins, fibers, polyphenols, flavonoids, and ascorbic, and these have aroused much interest for their role in lipid and antioxidant metabolism [3–7]. 

Several herbal combinations have been tested experimentally for their potential in ameliorating various ailments, for example, diabetes, allergic rhinitis, atherosclerosis, rheumatoid arthritis, and antimicrobial activity [7–12]. For instance, an experimental combination (of roots of ashwagandha, rhizomes of ginger, and young mulberry leaves) treatment given to NIDDM human subjects revealed a significant reduction in blood glucose, total cholesterol, triglycerides, LDL cholesterol and VLDL cholesterol [13]. 

A number of polyherbal formulations to treat various disorders are also being marketed by several reputed ayurvedic drug manufacturers in India which include among others Chyawanprash, Active Blood Purifier (44 and 7 herbs, resp.; Dabur India Ltd.), D-400, Liv 52, OST-6, Cystone (32, 7, 5, and 7 herbs, resp.; Himalaya Drug Co.), Livergen (6 herbs; Standard Pharmaceuticals), Livokin (17 herbs; Herbo-Med), Stimuliv (4 herbs; Franco-Indian Pharmaceuticals Pvt. Ltd.), Tefroliv (9 herbs; TTK Pharma Pvt. Ltd.), Pankajakasthuri (14 herbs; Herbals India Pvt. Ltd.), and Dihar (8 herbs; Rajsha Pharmaceuticals). 

The present investigation is aimed at finding an effective combination of different proportions of certain medicinal plants which could supplement as a therapeutic agent for hyperlipidaemia. The plants used in the present investigation, that is, *Glycyrrhiza glabra *(F. Fabaceae),* Withania somnifera *(F. Solanaceae), *Asparagus racemosus* (F. Liliaceae),* Chlorophytum borivilianum* (F. Liliaceae), and *Sesamum indicum* (F. Pedaliaceae), have ethnobotanical history and are widely used in Ayurvedic System of Medicine in India for various ailments. Our previous work on *G. glabra*, *C. borivilianum*,* W. somnifera*, *S. indicum*, and* A. racemosus* showed dose-dependent (5 and 10 gm% of roots/seed powder) beneficial effects on hypercholesterolemic rats [14–18]. We found that *G. glabra* was the most potent of the plants studied with reference to hypolipidaemic/hypocholesterolemic and antioxidant effects followed by* W. somnifera*, *A. racemosus*, *C. borivilianum*, and *S. indicum*. In this study, we have investigated the effect of different combinations of these plants on lipid profiles, fecal-cholesterol, neutral sterol, and bile acid excretion patterns and the consequent effects on hepatic cholesterol and bile acid production in hypercholesterolemic rats. An attempt was also made to study the status of hepatic lipid peroxidation, the profiles of antioxidants-catalase, superoxide dismutase, and ascorbic acid as well as ferric reducing ability of the plasma (FRAP) in hypercholesterolemic rats.

## 2. Results

### 2.1. Phytoconstituents and FRAP Value in Different Combinations

The quantitative phytochemical analysis of all three combinations (C-I, C-II, and C-III) indicated that these combinations contained fiber, phytosterols, saponin, polyphenol, flavonoids, and ascorbic acid. The FRAP values as total antioxidant concentrations in combinations I, II, and III indicated the highest value for C-I followed by C-II and C-III (Table 2).

### 2.2. Body Weight, Food Intake, and Liver Weight

There were no significant differences in food intake or body weights in any group (NC, HC, C-I, C-II, and C-III). However, liver weight decreased significantly (*P* < 0.002) in C-I (15%), C-II (14%), and C-III (12%) groups compared to HC animals (data not presented). 

### 2.3. Plasma and Hepatic Lipid Profiles

**Figure 1:**
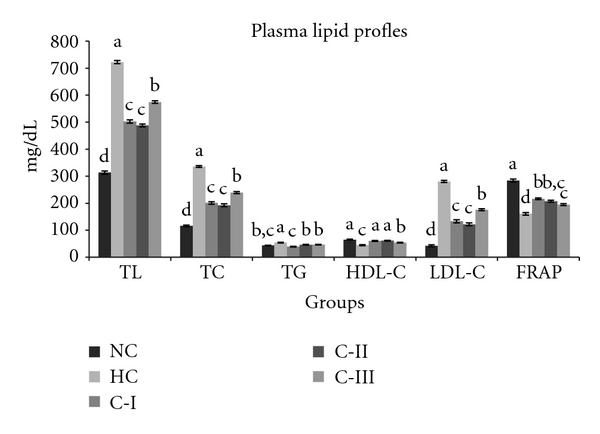
Effect of feeding different combinations on plasma lipid profiles (mg/dL). NC: normal controls; HC: hypercholesteremic animals; C-I: hypercholesterolemic animals administered with 5 gm% combination-I; C-II: hypercholesteremic animals administered with 5 gm% combination-II; C-III: hypercholesteremic animals administered with 5 gm% combination-III. Values: mean ± SEM and (*n* = 6); FRAP-value = (*μ*mol/Liter). Significant differences (*P* < 0.002) among the groups are denoted by a, b, c, and d.

The plasma lipid profiles significantly decreased (*P* < 0.002) with all three combinations. All three groups (C-I, C-II, and C-III) registered significant decline in plasma TL (C-I: 30%; C-II: 32%; C-III: 20%), TC (40%; 43%; 29%), TG (28%; 15%; 14%), and LDL (53%; 57%; 37%) levels, and an increase in HDL-C (35%; 37%; 21%, *P* < 0.002) was noted in these groups as compared to HC group (Figure 1). Very-low-density lipoprotein (VLDL) levels in the test groups (C-I: 7.8 ± 0.2; C-II: 9.2 ± 0.2; C-III: 9.3 ± 0.2 mg/dL, *P* < 0.002) were significantly lower than in HC group (10.8 ± 0.2 mg/dL). Thus, the combination diets reduced the VLDL concentrations by 28%, 15%, and 14%, respectively. Similarly, the atherogenic index (AI) was also reduced in the combination-diet-treated groups (C-I: 3.3 ± 0.1; C-II: 3.1 ± 0.1; C-III: 4.4 ± 0.1, *P* < 0.002) as compared to HC group (7.6 ± 0.3). The hepatic lipid profiles too significantly declined (*P* < 0.002) in the test groups: TL (by 28%, 34%, and 20% in C-I, C-II, and C-III, resp.) and TC (by 31%, 35%, and 20% in C-I, C-II, and C-III, resp.) but not significantly TG (by 17%, 9%, and 8% in C-I, C-II, and C-III resp., *P* < 0.002) as compared to HC group (Table 3).

### 2.4. Hepatic HMG-CoA Reductase and Bile Acids Levels

Administration of different combinations to hypercholesterolemic rats resulted in a significant increase in (*P* < 0.002) hepatic HMG-CoA reductase activity (C-I, C-II, and C-III groups: 16%, 19%, and 13%, resp.) as compared to HC group. Hepatic bile acid content also increased (*P* < 0.002, 27%; 33%; 16%) in these groups as compared to those of hypercholesterolemic control group (Table 3). 

### 2.5. Fecal Cholesterol, Neutral Sterols, and Bile Acid Content

There were increments (*P* < 0.002) in fecal cholesterol (15%; 19%; 11%), neutral sterol (19%; 23%; 12%) and bile acid (29%; 34%; 18%) excretion in C-I, C-II, and C-III groups as compared to HC group (Table 4). 

### 2.6. Hepatic Lipid Peroxidation and Antioxidant Profiles

**Figure 2:**
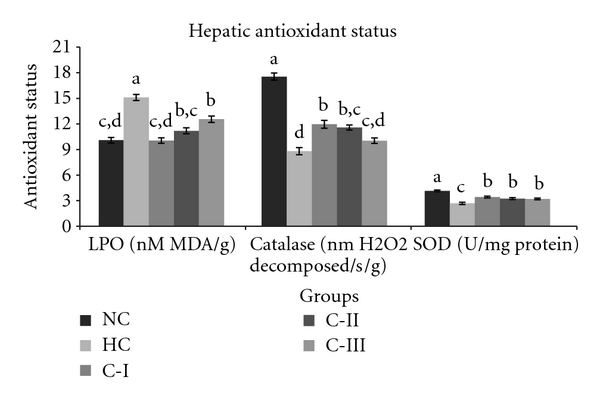
Effect of feeding different combinations on hepatic concentrations of lipid peroxidation (LPO), activities of catalase, and superoxide dismutase (SOD). Values: mean ± SEM and (*n* = 6). Significant differences (*P* < 0.002) among the groups are denoted by a, b, c, and d.

The hepatic lipid peroxidation decreased significantly (*P* < 0.002) in all three groups (C-I: 33%, C-II: 26%, and C-III: 17%) when compared to that of HC group. Both catalase (*P* < 0.002, C-I: 36%, C-II: 32%, and C-III: 14%) and SOD (*P* < 0.002, C-I: 27%, C-II: 20%, and C-III: 19%) activities also increased in these groups significantly compared to those in HC group (Figure 2). The C-I, C-II, and C-III group rats registered had increased hepatic ascorbic acid content (132.7 ± 3.8; 114.3 ± 3.1; 110.3 ± 3.2 *μ*g/g, *P* < 0.002) as compared to the hypercholesterolemic (95.5 ± 2.8 *μ*g/g) rats. FRAP values increased in all groups that is, C-I, CII, and C-III (*P* < 0.002, 35%; 29%; 21%, resp.), as compared to HC group (Figure 1).

## 3. Discussion

The present investigation demonstrates that the plant combinations used in this study are effective in lowering cholesterol levels in hypercholesterolemic rats. Plant metabolites, the sterols, soybean proteins, and fibers are reported to reduce the risk of CHD through their cholesterol lowering properties, independently and also in combination [8]. In this study, the observed cholesterol-lowering effects of different plant combinations administered to hypercholesterolemic rats could be related to an increased excretion of cholesterol, neutral sterols, and bile acid. The phytochemical analysis of the combinations (C-I, C-II, and C-III) revealed the presence of fibers, saponins, phytosterols, polyphenols, flavonoids, and ascorbic acid among which phytosterols, fibers as well as saponins could be important in cholesterol elimination in hypercholesterolemic rats administered with different combinations. These components have received considerable attention for their plasma cholesterol-lowering effects [3, 19–21]. It is known that phytosterols have a greater affinity for micelles than cholesterol because of their greater hydrophobicity. Therefore, they can easily displace intestinal cholesterol from the micelles, reducing intestinal cholesterol absorption, and consequently reduce hepatic and plasma cholesterol concentrations [21, 22]. Dietary fibers appear to interfere with cholesterol absorption and its enterohepatic bile circulation resulting in depletion of hepatic cholesterol pools and alteration in lipoprotein metabolism. Besides, the cholesterol lowering effect of fibers is considered primarily due to an increased excretion of cholesterol and bile acids [19, 23]. Saponins are also capable of precipitating cholesterol from micelles and interfering with enterohepatic circulation of bile acids making it unavailable for intestinal absorption and hence reduce plasma cholesterol levels [20, 24]. It is noteworthy that the excretion of cholesterol, neutral sterol, and bile acid was significantly higher with administration of different plant combinations when compared to that of hypercholesterolemic rats. These losses of fecal cholesterol and bile acid could have depleted hepatic cholesterol levels, and, as a consequence, a compensatory increase in hepatic cholesterogenesis and bile acid synthesis was noted with demonstrably higher hepatic HMG-CoA reductase activity and bile acid production in different combination groups as compared to those of hypercholesterolemic control rats. A significant decline in plasma LDL-cholesterol along with increased bile acid production in different combination groups could also be due to the fiber and saponin content of combinations as both fibers and saponins are reported to lower plasma LDL-cholesterol levels via interruption of cholesterol and bile acid absorption and increased LDL-receptor activity; the LDL-cholesterol could then be subsequently catabolized to bile acid [23, 25].

 Rats fed different combinations had lower hepatic and plasma TG concentrations than rats fed cholesterol alone. The TG depletion may be related to the ability of fiber to inhibit absorption of lipids from the intestinal lumen [23]. Moreover, saponins are reported to inhibit pancreatic lipase activity and reduce the TG concentrations [26] leading to subsequent reduction in the VLDL-C concentration in plasma [22]. Presently all three experimental groups (C-I, C-II and C-III) showed a simultaneous decline in plasma TG and VLDL-C levels that could be correlated with the fiber and saponin content of the experimental diets. Several studies documented that low levels of HDL-cholesterol are indicative of high risk for CHD and an increase in HDL-C level is considered beneficial [1]. Epidemiological studies have also shown that high HDL-cholesterol levels could potentially contribute to antiatherogenecity, inhibit LDL-oxidation and protect endothelial cells from the cytotoxic effects of oxidized LDL [27]. Presently noted high levels of plasma HDL-cholesterol in hypercholesterolemic animals administered with different combinations as compared to HC animals indicate the physiological role played by these combinations in elevating HDL-cholesterol levels. While dietary saponins and fibers are not known to elevate HDL-cholesterol levels [20, 23], ascorbate and flavonoids are reported to increase the HDL-cholesterol concentrations [28]. As the combinations contained both ascorbic acid and flavonoids, the increased HDL-cholesterol levels in hypercholesterolemic animals administered with combination diets could be attributed to these plant metabolites. 

Recent studies have demonstrated a direct relationship between hypercholesterolemia and CHD [1]. The dietary cholesterol during its metabolism is delivered to the hepatic cells where substantial amounts of reactive oxygen species are generated [29]. This process is believed to generate highly toxic products, including lipid peroxides as aldehydes, epoxides and carbonyls, and cause rapid consumption of antioxidants such as vitamin E or vitamin C [29]. Further, high cholesterol diet increases serum LDL levels, and, due to oxidative stress, the LDL is oxidized increasingly thereby facilitating atherosclerotic plaque formation [30]. Many studies have shown that dietary polyphenols, flavonoids, carotenoids, vitamin C, and lignans can effectively scavenge free radicals, reduce the levels of lipid peroxidation and oxidation of LDL and, prevent the progress of atherosclerosis [3, 28, 30–32]. The polyphenols with their antioxidant activity, chiefly due to their redox properties, act as reducing agents, hydrogen donors, and singlet oxygen quenchers with a metal chelating potential [3, 32]. The polyphenols and flavonoids are reported to scavenge free radicals, including hydroxyl and superoxide anions, stimulate SOD and catalase gene expression, reduce the malondialdehyde concentrations, and improve lipid profiles in experimental animals [10, 32, 33]. Ascorbic acid is a major chain-breaking antioxidant against free radicals and conjugates with cytotoxic, genotoxic, and lipid peroxidation products to eliminate them [34]. The root extract of *G. glabra* has antioxidant activity reportedly due to the presence of a variety of phenolic compounds including flavonoids, isoflavonoids, chalcones, and bibenzyls [35]. The antioxidant and antiperoxidative properties of sesame seed are related to lignans, such as sesamol, sesamolinol, pinoresinol, and sesaminol [31]. The sitoindosides VII-X and Withaferin A (Glycowithanolides) of *W. somnifera* have been implicated in increasing the antioxidant activity in rat brain and stimulated catalase and SOD activities [36]. In the present study, high cholesterol (HC) fed rats showed a remarkable decline in antioxidant status and concurrently increased lipid peroxidation as compared to the normal control (NC) rats. However, all three combinations treated hypercholesterolemic rats indicate considerable decrease in lipid peroxidation and improvement in the antioxidant status. Further, the aqueous extract and plasma FRAP-values as well as hepatic catalase, SOD and ascorbic acid levels of C-I were highest compared to C-II and C-III groups. The malondialdehyde level in C-I group also showed a significant decline as compared to C-II and C-III groups. The phytochemical analyses of combinations have indicated the presence of polyphenols, flavonoids, and ascorbic acid. Consequently, these elevated levels of hepatic antioxidants in combinations-treated rats might be due to the presence of polyphenols and flavonoids in the experimental diet. In this context, our recent in vitro studies on aqueous and ethanolic extracts of *G. glabra, A. racemosus, C. borivilianum, *and *S. indicum* demonstrated that these plants possess considerable free radical scavenging potency against superoxide, nitric oxide, and hydroxyl radicals due to phenolic content of plants [37–40]. In addition, these plants markedly suppressed the metal-catalyzed lipid peroxidation in hepatic mitochondrial fractions isolated from rats as well as favorably affected atherosclerosis risk status by reducing human serum and LDL oxidation caused by copper ion [37–40]. Taken together, presently observed high antioxidant status in combinations-treated hypercholesterolemic rats may be due to free radical scavenging action exerted by combinations diet. Additionally, the combinations could be a potent source for ascorbic acid as it suppressed the propagation of lipid peroxidation and reduced the levels of malondialdehyde. Unlike our earlier reports on these individual plants [14–18], the combinations used in the present investigation (C-I, C-II, and C-III) are with much lower levels of additions of individual plants (ranging between 0.625 and 1.5 gm as against 5 and 10 gm%) in the diet (Table 1). Yet these combinations significantly reduced the lipid profiles and improved the body antioxidant capacity of hypercholesterolemic rats. Thus, the herbal combinations-C-I, C-II, and C-III, exhibited differential hypocholesterolemic and antioxidant activities when fed to hypercholesterolemic rats. This could be due to varying concentrations of different classes of phytocomponents in the combinations such as fiber, phytosterols, saponins, polyphenols, flavonoids and vitamin C (Table 2). In conclusion, although all the three tested combinations of plants were effective as hypocholesterolemic and antioxidant agents, C-I appeared to be more potent in reducing body lipid profiles and lipid peroxidation and improving the antioxidant status of hypercholesterolemic rats.

## 4. Materials and Methods

### 4.1. Preparation of Plant Combinations and Phytochemical Analysis

The roots of *Glycyrrhiza glabra *(GG) and seeds of *Sesamum indicum* (SI) were purchased from the local merchandise and roots of *Withania somnifera *(WS)*, Asparagus racemosus *(AR) and* Chlorophytum borivilianum* (CB) were collected from University Botanical Gardens and authenticated by our faculty Taxonomist Dr. A. S. Reddy. Roots and seeds were air-dried completely, ground to powder, and used for preparation of different combinations (i.e., combinations I, II, and III). All combinations were prepared using a 5 gm% mixture of roots/seed powder of the plants (Table 1). 

Phytoconstituents of C-I, C-II, and C-III were quantified by standard methods. The total fiber, polyphenol, and flavonoid were estimated according to Thimmaiah [41]. Phytosterol and saponin contents of the combinations were estimated using ferric chloride-sulphuric acid and vanillin-sulphuric acid methods, respectively, with *β*-sitosterol and saponin as standards [42, 43]. The total ascorbic acid content was estimated using 2,4-dinitrophenyl hydrazine reagent [44]. The ferric-reducing ability of combinations (FRAP) were measured as the concentrations of total antioxidants using the TPTZ (2,4,6-tripyridyl-*s*-triazine) HCl-FeCl_3_ reagent at 593 nm. Calibration curve of ferrous sulfate was used [45]. 

### 4.2. Animals

Male albino rats (Charles Foster, bred in the Department's Animals House, weighing 150–200 gm) were housed individually with *ad libitum* access to water and fed on commercial feed (Pranav Agro Ind. Ltd., Pune, India) in a well-ventilated animal unit (26 ± 2°C, humidity 56%, 12 hour-light/dark cycle). The care and procedures adopted for the present investigation were in accordance with rules and regulations of CPCSEA, and the experiment was approved by Institutional Animal Ethics Committee (IAEC).

### 4.3. Experimental Design

After a 10-day adaptation period, 30 rats were divided into 5 groups of 6 rats each: NC: normal control rats receiving only commercial feed/ basal diet, HC: high cholesterol diet control rats receiving basal diet with 0.5 gm% cholesterol and 1.0 gm% sodium taurocholate, C-I: rats receiving high cholesterol diet + combination I, C-II: rats receiving high cholesterol diet + combination II, and C-III: rats receiving high cholesterol diet + combination III. The basal diet contained 55.67 gm% carbohydrates, 22.12 gm% protein, 4.06 gm% fat, 3.76 gm% fiber, 5.64 gm% mineral mixture, and 8.75% moisture content. 

At the end of the four-week treatment, 24-hour fecal samples were collected from individual cages. Animals were fasted overnight and sacrificed under mild anesthesia (diethyl ether). Blood was collected by cardiac puncture, and plasma was separated by centrifugation. Liver was excised, and, both plasma and liver were kept frozen until analyzed.

### 4.4. Plasma and Hepatic Lipid Profiles

Plasma total lipid (TL) content was estimated by sulpho-phosphovanillin method [46]. Plasma cholesterol (TC), HDL-cholesterol (HDL-C), and triglycerides (TG) were assayed using commercial diagnostic kits (Eve's Inn Diagnostics, Vadodara, India). Low-density lipoprotein cholesterol (LDL-C), very-low-density lipoprotein cholesterol (VLDL-C), and atherogenic index (AI) were calculated according to Friedewald equations [47]. The liver TL was extracted in chloroform : methanol (2 : 1) [48] and estimated gravimetrically. Hepatic TC and TG were extracted [48] and estimated using the standard kits (Eve's Inn Diagnostics, Vadodara, India). 

### 4.5. Hepatic HMG-CoA Reductase and Bile Acid Profile

Hepatic HMG-CoA reductase (EC 1.1.1.34) activity was measured in terms of the ratio of HMG-CoA to mevalonate using hydroxylamine-FeCl_3_ reagent [49]. Briefly, the hepatic HMG-CoA was determined by its reaction with hydroxylamine reagent at alkaline pH and followed by colorimetric measurement of the resulting hydroxamic acid by formation of complexes with ferric salts at 540 nm. The hepatic mevalonate was estimated by reaction with the same reagent but at pH 2.1. At this pH, the lactone form of mevalonate readily reacts with hydroxylamine to form the hydroxamate. Thus, hepatic HMG-CoA and mevalonate concentration in the tissue homogenate are estimated in terms of absorbances, and the ratio between the two is taken as an index of the HMG-CoA reductase enzyme activity that catalyzes the conversion of 3-hydroxy-3-methylglutaryl-coenzyme A to mevalonate. The ratio is inversely proportional to the enzyme activity, that is, the increase in ratio corresponds to a decrease in enzyme activity. The alkaline-ethanol extract of hepatic bile acid was acidified and estimated using vanillin-phosphoric acid reagent [50]. 

### 4.6. Fecal Cholesterol, Neutral Sterol, and Bile Acid Content

The fecal cholesterol, neutral sterol, and bile acid were extracted using alkaline methanol medium [51], and fecal cholesterol and neutral sterol contents were estimated [52] using standard kits (Eve's Inn Diagnostics, Vadodara, India). A portion of the extract was acidified and used for bile acid estimation [50]. 

### 4.7. Hepatic Lipid Peroxidation and Antioxidant Profile

The concentration of the lipid peroxide products of hepatic TBARS (Thiobarbituric acid reactive substances) was measured by TBA-TCA-HCl reagent (0.37% TBA, 15% TCA, 0.25 N HCl) at 532 nm using a molar absorption coefficient of 1.56 × 10^5^ M^−1^cm^−1^ [53]. Catalase (EC 1.11.1.6) activity was measured spectrophotometrically as decomposition of H_2_O_2_ at 240 nm [54]. The superoxide dismutase (SOD; EC 1.15.1.1) was assayed by using NADPH and PMS (phenazine methosulfate) reagents under nonacidic conditions, which reduces NBT (nitroblue tetrazolium salt) and forms a blue coloured formazon that can be measured at 560 nm [55]. Total ascorbic acid was determined using 2,4-dinitro phenyl hydrazine (DNPH) H_2_SO_4_ reagent [44]. The FRAP value of plasma was measured by the method of Benzie and Strain [45]. 

### 4.8. Statistical Evaluation

Results are expressed as means ± SEM. Significant differences among the groups were determined by one-way ANOVA using the 10th version of SPSS with Duncan's test as post hoc analysis. Differences were considered significant if *P* < 0.002.

##  Conflict of Interests

The authors declare that they have no conflict of interests.

## Figures and Tables

**Table 1:** Combinations of different plants (5 gm%) used.

Plants	C-I (gm)	C-II (gm)	C-III (gm)
*Glycyrrhiza glabra*	1.5	0.625	1
*Withania somnifera*	1.25	0.75	1
*Asparagus racemosus*	0.875	0.875	1
*Chlorophytum borivilianum*	0.75	1.25	1
*Sesamum indicum*	0.625	1.5	1

**Table 2:** Phytoconstituents of feed combinations (triplicate values: mean ± SD).

Phytoconstituents mg/gm dry tissue	Combination I	Combination II	Combination III
Fiber	132.66 ± 1.15 (13.266%)	125.66 ± 1.15 (12.566%)	102 ± 1.73 (10.2%)
Phytosterols	13.13 ± 0.34 (1.313%)	16.31 ± 0.65 (1.631%)	10.57 ± 0.67 (1.057%)
Saponins	52.60 ± 1.18 (5.260%)	59.10 ± 1.23 (5.910%)	42.96 ± 1.05 (4.296%)
Polyphenols	16.17 ± 1.15 (1.617%)	12.65 ± 0.87 (1.265%)	10.37 ± 0.62 (1.037%)
Flavonoids	4.39 ± 0.42 (0.439%)	3.07 ± 0.27 (0.307%)	2.61 ± 0.41 (0.261%)
Ascorbic acid	5.87 ± 0.38 (0.587%)	3.98 ± 0.38 (0.398%)	3.29 ± 0.34 (0.329%)
FRAP value mmole/gm	0.045 ± 0.003	0.04 ± 0.002	0.031 ± 0.003

**Table 3:** Effect of feeding different combinations on hepatic lipid profiles, HMG-CoA reductase activity and bile acid content (mg/gm).

Group	NC	HC	C-I	C-II	C-III
Total lipids	30 ± 2.9^d^	126.8 ± 4.3^a^ (+322.76)	91.7 ± 3.1^bc^ (−27.72)	83.8 ± 3.2^c^ (−33.90)	101.3 ± 3.1^b^ (−20.10)
Total cholesterol	1.9 ± 0.1^d^	36.1 ± 1.7^a^ (+1769.43)	24.9 ± 1.5^bc^ (−30.90)	23.5 ± 1.3^bc^ (−34.75)	29 ± 1.4^b^ (−19.53)
Triglycerides	3 ± 0.1^b^	24.3 ± 1.4^a^ (+705.96)	20.2 ± 1.2^a^ (−17.00)	22.1 ± 1.7^a^ (−9.20)	22.3 ± 1.8^a^ (−8.50)
HMG CoA reductase*	2.8 ± 0.1^e^	7.1 ± 0.1^a^ (−156.67)	6 ± 0.1^bc^ (+15.75)	5.8 ± 0.1^cd^ (+18.84)	6.2 ± 0.1^b^ (+12.93)
Bile acid	3.8 ± 0.1^d^	6.9 ± 0.1^c^ (+83.55)	8.8 ± 0.1^a^ (+27.45)	9.2 ± 0.1^a^ (+33.09)	8 ± 0.1^b^ (+16.18)

Values: mean ± SEM (*n* = 6).

Figures in parentheses indicate percent increase (+) or decrease (−).

Within a row those with different superscripts are significantly different (*P* < 0.002).

*HMG-CoA reductase activity is inversely proportional to the ratio of HMG-CoA/mevalonate (absorbance of HMG-CoA/absorbance of mevalonate), that is, the increase in ratio corresponds to a decrease in enzyme activity.

Comparisons for the percentage were made between groups NC and HC; HC and C-I; HC and C-II; HC and C-III.

**Table 4:** Effect of feeding different combinations on fecal cholesterol, neutral sterols, and bile acid excretion (mg/gm).

Group	NC	HC	C-I	C-II	C-III
Cholesterol	1.9 ± 0.1^d^	6.1 ± 0.2^c^ (+208.08)	7 ± 0.1^ab^ (+15.24)	7.2 ± 0.1^ab^ (+18.85)	6.8 ± 0.2^bc^ (+10.98)
Neutral sterols	4.9 ± 0.2^d^	8.2 ± 0.2^c^ (+66.26)	9.8 ± 0.1^ab^ (+19.19)	10.1 ± 0.2^a^ (+23.08)	9.2 ± 0.2^b^ (+12.39)
Bile acid	5.7 ± 0.2^c^	11 ± 0.4^b^ (+94.34)	14.2 ± 0.5^a^ (+29.18)	14.7 ± 0.3^a^ (+33.90)	13 ± 0.3^a^ (+18.36)

Values: mean ± SEM (*n* = 6).

Figures in parentheses indicate percent increase (+) or decrease (−).

Within a row those with different superscripts are significantly different (*P* < 0.002).

Comparisons for the percentage were made between groups NC and HC; HC and C-I; HC and C-II; HC and C-III.
